# Chloroplast genome comparative analysis and phylogenetic relationships of 15 *Syringa* species (Oleaceae)

**DOI:** 10.1590/1678-4685-GMB-2026-0016

**Published:** 2026-09-07

**Authors:** Yuting Duan, Yan Wang, Yiluo Wang, Kaige Xu, Erdong Zhang, Xiaodong Ding, Lei Zhang

**Affiliations:** 1 North Minzu University, School of Biological Science & Engineering, Key Laboratory of Ecological Protection of Agro-Pastoral Ecotones in the Yellow River Basin, National Ethnic Affairs Commission of the People’s Republic of China, Yinchuan, China.; 2 North Minzu University, Analysis and Testing Center of Ningxia Hui Autonomous Region, Yinchuan, China.

**Keywords:** Syringa, chloroplast genome, comparative analysis, phylogenetic relationship, divergence time

## Abstract

*Syringa* is a crucial shrub genus in the family Oleaceae, which has significant ornamental, economic, and medicinal value. However, research on the chloroplast genome (CPG) phylogeny and lineage diversification of this genus remains limited. In this study, all 15 *Syringa* CPGs exhibited a characteristic quadripartite structure, with genome lengths ranging from 154,019-158,020 bp. These CPGs were highly conserved and moderately differentiated, each containing 130-132 genes. Analysis of inverted repeat (IR) boundaries indicated structural conservation, with six genes: *rps*19, *rpl*2, *ycf*1, *trn*N, *ndh*F, and *trn*H present at the IR/single-copy (SC) junctions. The small single copy (SSC) region displayed greater sequence variability than the IR regions. *ycf*1, *ndh*H, *trn*L-*rpl*32, *ndh*F-*ycf*1, and *rbc*L-*acc*D were identified as potential molecular markers and *rps*11, *ycf*2, and *ycf*4 may have contributed to the adaptive evolution of *Syringa*. Phylogenetic reconstruction based on whole CPG data supported the monophyly of the 15 species, which were divided into three distinct subclades. Molecular dating estimated that *Syringa* diverged from its sister genus approximately 58 million years ago, with most *Syringa* species diversifying further approximately 47.49 million years ago during the Eocene. Our findings will hopefully stimulate further studies on this genus that may enhance biodiversity knowledge.

## Introduction

*Syringa* is a vital shrub genus within the Oleaceae family and comprises approximately 40 species distributed across Asia, including China, North Korea, and Japan as well as southeastern Europe. Within China, it is primarily native to southwestern regions and the provinces along the Yellow River (Chang et al., 1996; Wang et al., 2022). *Syringa* are not only valued as ornamentals plants but also offer economic benefits and significant medicinal properties (Zhu et al., 2021). They have been cultivated for centuries in China and Europe as decorative plants and sources of spice (Fiala and Vrugtman, 2008). Numerous studies have shown that these plants contain bioactive compounds such as terpenoids, which exhibit a range of therapeutic effects, including cardioprotective, neuroprotective, hypoglycemic, anti-influenza, antibacterial, anti-inflammatory, and antioxidant properties (Gao et al., 2020; Ma et al., 2020; Xu et al., 2025). Previous research on *Syringa* has primarily focused on their taxonomy (Qian et al., 2025), physiology (Xiao et al., 2015), breeding (Lattier and Contreras, 2017), and medicinal attributes (Zhang et al., 2014; Filipek et al., 2019; Peng et al., 2019). Despite their ornamental and medicinal significance, the key aspects of *Syringa* phylogeny, interspecific relationships, and evolutionary history remain poorly understood (Yang et al., 2023). Although researchers have explored the phylogenetic relationships within the genus using limited ITS and DNA barcode fragments (Li et al., 2002; Li *et al*., 2012; Cheng et al., 2025), the complete chloroplast genome (CPG) phylogeny and lineage diversification remains to be investigated.

Chloroplasts, are essential organelles in plants, which serve as the primary site of photosynthesis and are involved in critical processes such as carbon fixation (Qin et al., 2023; Li et al., 2025). They contain their own independent genetic system: the CPG, which is double-stranded and circular in most plants (Xie et al., 2020; Kassem, 2025). Compared with short DNA fragments, CPGs provide substantially more informative sites, which makes them particularly effective for analyzing nucleotide diversity and reconstructing phylogenetic relationships among closely related species (Daniell et al., 2016; Wang et al., 2023 a ; Qin *et al*., 2025). CPGs range between 75 and 250 kb in length, with numerous copies per cell, and are generally maternally inherited in most plant species. Their structure is highly conserved in terms of both gene content and organization (Wang *et al*., 2023b; Shi et al., 2025). A typical CPG comprises four distinct regions: two single-copy regions: large single copy (LSC) and small single copy (SSC) and a pair of inverted repeat regions (IR):IRa and IRb (Ruf et al., 2019; Guo et al., 2025). CPGs have become valuable tools in comparative genomic and phylogenetic studies due to their structural simplicity, high conservation, and uniparental inheritance. They are increasingly being used to identify species, assess genetic and nucleotide diversity, resolve complex phylogenetic relationships, and infer evolutionary history (Nock et al., 2019; Quan et al., 2024).

Despite the availability of CPG data, few molecular phylogenetic studies have utilized to resolve infrafamilial relationships within *Syringa*. Existing studies have largely relied on one or a few molecular loci, or limited taxonomic coverage. Consequently, phylogenetic relationships and divergence timescales within the genus remain poorly resolved (Li et al., 2002, 2012; Yang et al., 2023). In this study, we compared and analyzed 15 *Syringa* species. Our primary aims were to (1) reconstruct a robust phylogeny of the Oleaceae family using CPGs from 35 species, (2) estimate the divergence times of the *Syringa* clade, and (3) investigate the structural variations in CPGs across the sampled *Syringa* species.

## Material and Methods

### Plant material, DNA extraction, and sequencing

Thirty-seven CPGs from Oleaceae and related families were analyzed in this study (Table S1). Of these, 35 CPGs were selected from Oleaceae, including 15 species of *Syringa*, and two additional CPGs from Lamiaceae were included as outgroups for phylogenetic reconstruction. Among the 37 CPGs, *S. pinnatifolia*, *S. vulgaris*, and *S. pubescens* were newly sequenced in this study and the remaining sequences were retrieved from the National Center for Biotechnology Information (NCBI; https://www.ncbi.nlm.nih.gov/: Table S1). Leaf samples were collected from various locations throughout China. All plant materials were identified by Dr. Lei Zhang and voucher specimens were deposited in the Herbarium of North Minzu University (NMU; Yinchuan, China: Table S1). Total DNA was extracted from the silica gel-dried leaves using the cetyltrimethylammonium bromide (CTAB) method (Allen et al., 2006). Sequencing libraries were prepared using the NEBNext DNA Library Kit (New England Biolabs, Ipswich, MA, USA) according to the manufacturer’s protocol. The DNA was randomly fragmented into approximately 350-bp inserts, and paired-end sequencing (150 bp) was performed using a NovaSeq 6000 platform (Illumina, San Diego, CA, USA).

### Genome sequencing, assembly, and annotation

A minimum of two of 150-bp paired-end reads were generated per sample. Raw reads were filtered to remove those with quality scores below 7 or those containing more than 10% ambiguous nucleotides, thereby producing high-quality clean reads for downstream analyses. The filtered reads were assembled using NOVOPlasty v4.3.3 (Dierckxsens et al., 2017) with parameters set as k-mer = 39, read length = 150, and insert size = 350. The resulting contigs were aligned and merged into complete sequences using Geneious v11.0.3 (Kearse et al., 2012). The CPGs were annotated in Plann v1.1 (Huang and Cronk, 2015) using the *S.* × *persica* CPG as a reference. Finally, circular genome maps were generated using OGDRAW v1.2 (Lohse et al., 2013) and all newly annotated CPGs were submitted to GenBank (Sayers et al., 2020).

### Genomic structural and divergent hotspot analysis

Structural variations and rearrangement events were analyzed across 15 *Syringa* CPGs. Comparative analysis of these CPGs was performed using the mVISTA program (Frazer et al., 2004), with the annotated CPG of *S.* × *persica* serving as a reference in the LAGAN alignment mode (Brudno et al., 2023). Following sequence alignment, nucleotide diversity (Pi) across the CPGs was calculated using DnaSP version 6.0 (Rozas et al., 2017).

### Selection pressure analysis

We excluded protein-coding genes (PCGs) shorter than 300 bp. A total of 49 PCGs were chosen and aligned using the MUSCLE algorithm (Edgar, 2004). Stop codons were removed from the aligned sequences using MEGA 11 (Tamura et al., 2021). The resulting aligned sequences were converted to the PML format using PhyloSuite v1.2.2 (Zhang et al., 2020 a ). To calculate the non-synonymous substitution rate (Ka), synonymous substitution rate (Ks), and ratio values (ω, Ka/Ks) of each PCG, we employed the Site Model (M0) using PAML v4.9 (Yang, 2007).

### Phylogenetic inference and estimation of divergence time

Two datasets were constructed for phylogenetic reconstruction: a PCG set and a whole plastome set. PCGs were extracted from the 34 CPGs in GenBank format using custom Perl scripts, with the start and stop codons removed. After excluding potential pseudogenes, only the PCGs common to all species were retained. Each gene was aligned using PRANK v130410 (Löytynoja and Goldman, 2008) based on the translated amino acid sequences. Genes that were absent from any species were discarded and the remaining aligned sequences were concatenated into a supermatrix.

Phylogenetic analyses were conducted separately for the PCG and whole plastome datasets using both maximum likelihood (ML) and Bayesian inference (BI) approaches. ML analysis was performed using RAxML v8.1.24 (Stamatakis, 2014) under the GTR + Γ model, with the best tree identified via the rapid hill-climbing algorithm, and node support was assessed with 1,000 bootstrap replicates. The optimal substitution model (GTR + I + G) was selected using jModeltest, and BI was performed using MrBayes v3.2.6 (Ronquist et al., 2012). The resulting phylogenies were visualized using FigTree v1.4.2 (Rambaut, 2012).

Divergence times were estimated from the plastome dataset using the MCMC tree in PAML v4 (Yang, 2007) under a relaxed-clock model and birth-death sampling process (Rannala and Yang, 2007). As fossil records for Oleaceae are limited, four secondary time constraints were applied: (1) 11.7-22.5 million years ago (Mya) for the split between *Nestegis apetala* and *Notelaea microcarpa* (Dupin et al., 2022); (2) 5.78-14.3 Mya for the divergence of *Forestiera isabelae* and *Hesperelaea palmeri* (Li et al., 2019); (3) 19.9-38.3 Mya for the split between *Fraxinus chinensis* and related species (Zhang et al., 2020 b ); (4) 98.2-105 Mya for the root of the phylogeny, following previously established constraints (Roalson and Roberts, 2016).

The GTR + Γ model was used, with the prior for the substitution rate (rgene) set to a gamma distribution Γ(2, 200, 1). The birth-death process was parameterized with species sampling and σ² values set to (1, 1, 0.1) and G (1, 10, 1), respectively. We ran two independent MCMC chains, where the first 2,000 generations were discarded as burn-in; subsequently, and the output was sampled every 750 generations until 20,000 samples were obtained. Convergence was confirmed by comparing the two runs with random seeds, which yielded consistent results.

### Ethics

The current investigation involved the collection of *S. pubescens*, *S. vulgaris*, and *S. pinnatifolia* specimens from publicly accessible land. Such collection for research purposes does not pose a threat to the local ecology. All voucher specimens were deposited in the Herbarium of North Minzu University (NMU), Yinchuan, Ningxia, People’s Republic of China, under the voucher numbers zlnmu2025100, zlnmu2021150, and zlnmu2024085, respectively. Taxonomic identification was confirmed by Dr. Lei Zhang.

## Results

### Characteristics of *Syringa* CPGs

**Figure 1 -:**
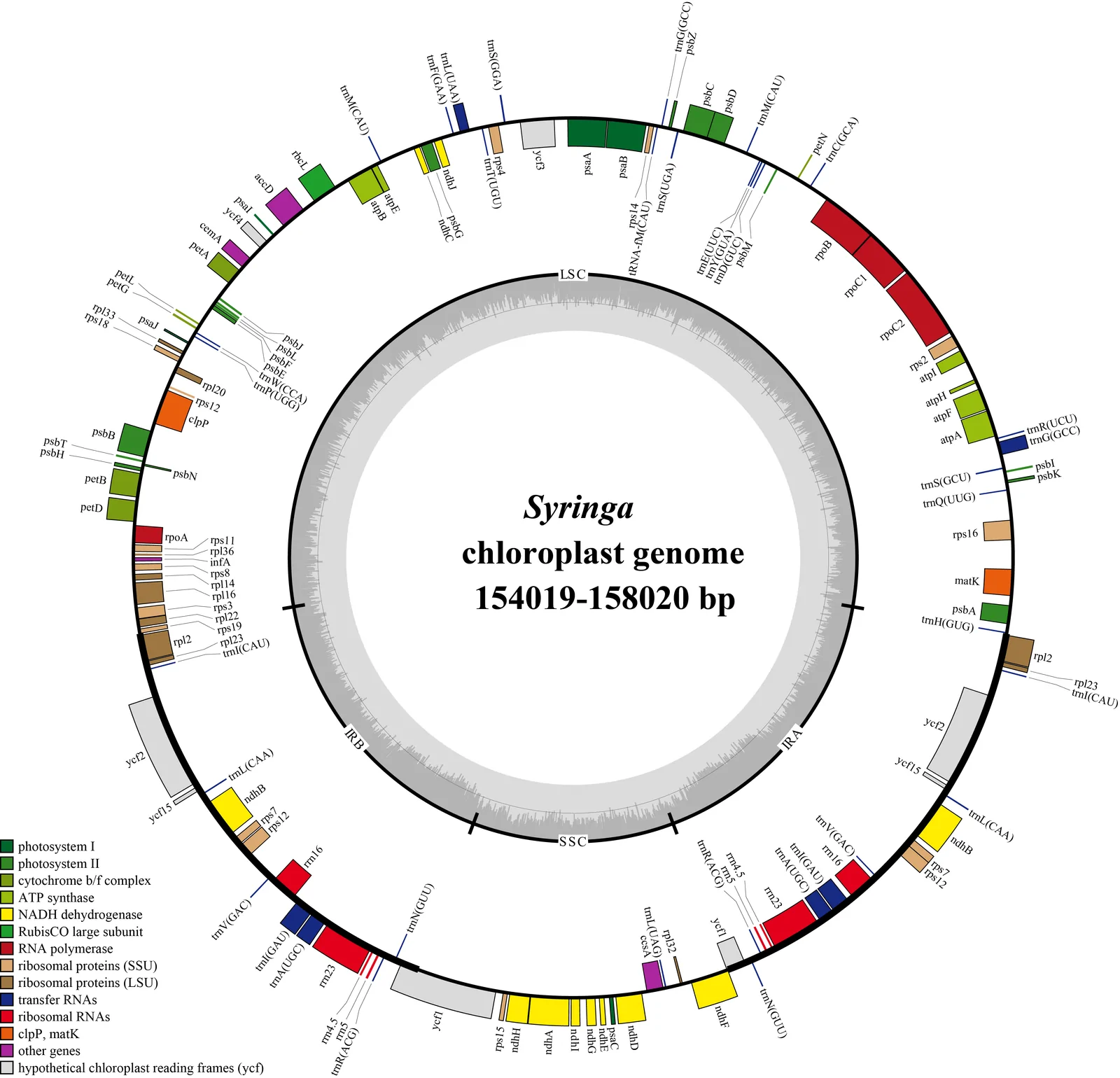
Gene map of the CPGs of 15 *Syringa* species. Genes belonging to different functional groups are shown in different colors. The darker gray area in the inner circle indicates the GC content, and the lighter gray indicates the AT content of the genome. The thick lines indicate the extent of the inverted repeats (IRa and IRb) that separate the genomes into the small single-copy (SSC) and large single-copy (LSC) regions.

Following quality control and preprocessing, a minimum of 6 Gb of whole-genome sequencing data were obtained for *S. pinnatifolia*, *S. pubescens*, and *S. vulgaris*. Clean reads were used to assemble complete CPGs through a reference-guided approach. All 15 *Syringa* CPGs exhibited their characteristic quadripartite circular structure consisting of LSC and SSC regions, separated by a pair of IR regions (Figure 1; Table S2). The total size of the CPGs ranged from 154,019 (*S. pubescens*) to 158,020 bp (*S. komarowii* subsp. *reflexa*). The LSC region varied in length from 86,213 (*S. pubescens*) to 87,108 bp (*S. reticulata* subsp. *amurensis*), the SSC region ranged from 17,239 (*S. reticulata* subsp. *amurensis*) to 19,245 bp (*S. pinetorum*), and the IR regions from 25,046 (*S. pubescens*) to 25,897 bp (*S. reticulata* subsp. *amurensis*) (Table S2). The GC content was highly conserved across all *Syringa* CPGs, and ranged from 37.9% to 38.0%. The IR regions showed higher GC content (42.9-43.4%) than the LSC (31.1-36.2%) and SSC (32.1-32.9%) regions (Figure 1; Table S2). Additionally, each CPG contained 130-132 annotated genes, including 35-37 tRNAs and eight rRNAs (Table S2).

### IR boundary analysis

**Figure 2 -:**
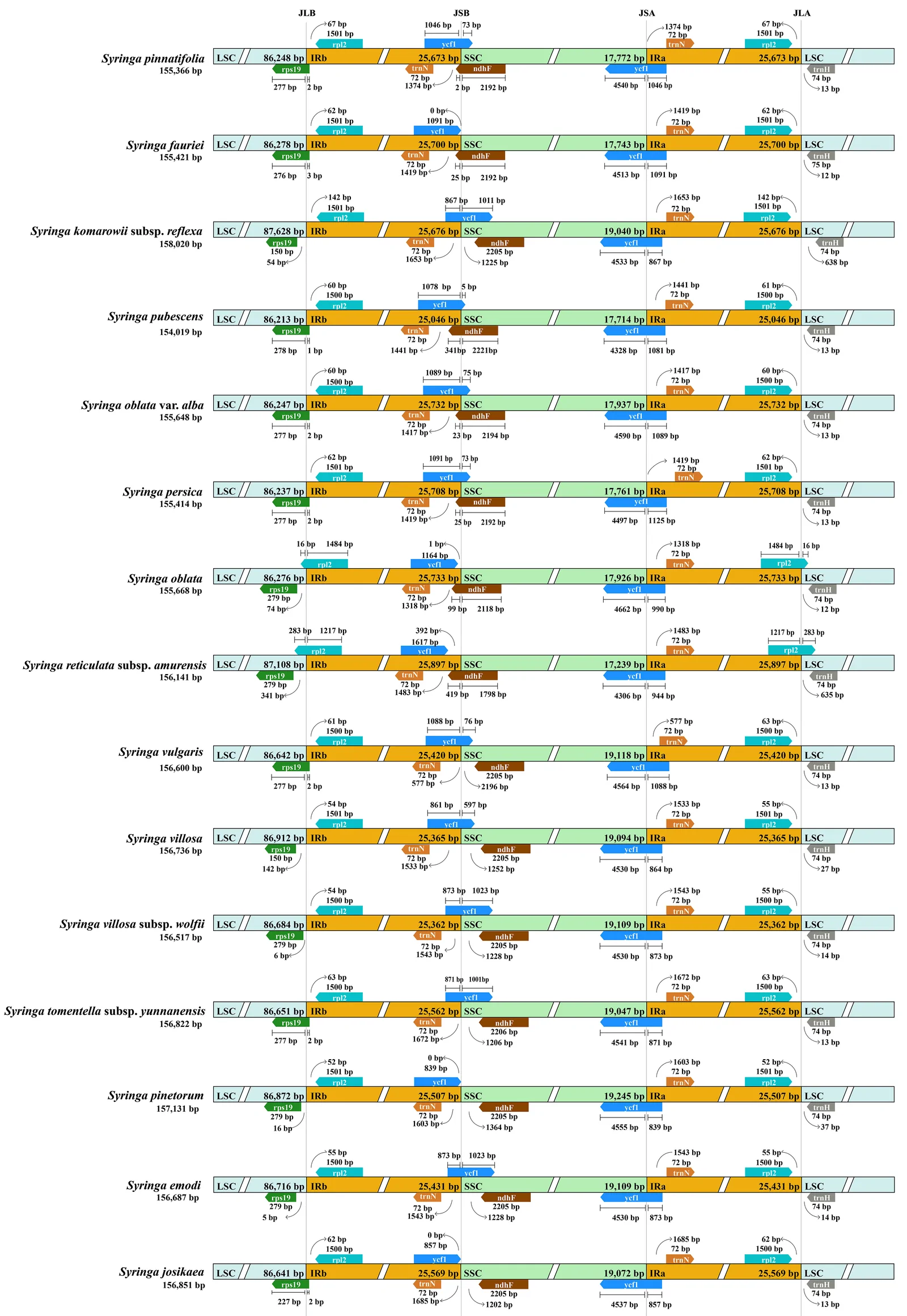
Comparisons of the borders of the large single-copy (LSC), small single-copy (SSC), and inverted repeat (IR) regions among the CPGs of 15 *Syringa* species.

The IR/single copy (SC) boundary regions were compared across the 15 *Syringa* species (Figure 2). The CPGs showed high structural conservation, particularly in the size of the IR regions (25,420-25,897 bp) and organization of the IR/SC boundaries. Six genes were consistently present near these boundaries: *rps*19 and *rpl*2 between the LSC and IRb; *ycf*1, *trn*N, and *ndh*F between the IRb and SSC; *ycf*1 and *trn*N between the SSC and IRa; and *rpl*2 and *trn*H between the IRa and LSC. Notable variations were observed in the positioning of specific genes: The *rpl*2 gene spanned the LSC/IRb and IRa/LSC boundaries in *S. oblata* and *S. reticulata* subsp. *amurensis* but were entirely contained within the IR region in other species. *rps*19 spanned the LSC/IRb boundary in seven species, extending 1-3 bp into the IRb; in others, it resided entirely in the LSC. The SSC/IRb boundary was generally situated within *ycf*1 and *ndh*F, except in *S. fauriei*, *S. oblata*, *S. pinetorum*, *S. josikaea*, and *S. reticulata* subsp. *amurensis*. The *ycf*1 gene extended into the SSC by 5 bp (*S. pubescens*) to 1,023 bp (*S. villosa* subsp. *wolfii*), whereas *ndh*F was located entirely in the SSC in eight species. At the SSC/IRa boundary, *ycf*1 consistently marked this transition. Further, *trn*N was invariably located within the IR region, 577-1,685 bp from the nearest boundary and *trn*H was consistently present in the LSC at 12-638 bp from the IRa/LSC boundary. These results reflect both conserved features and lineage-specific structural variations in the IR/SC boundaries of *Syringa* CPGs.

### Comparative genomics and divergence hotspots

**Figure 3 -:**
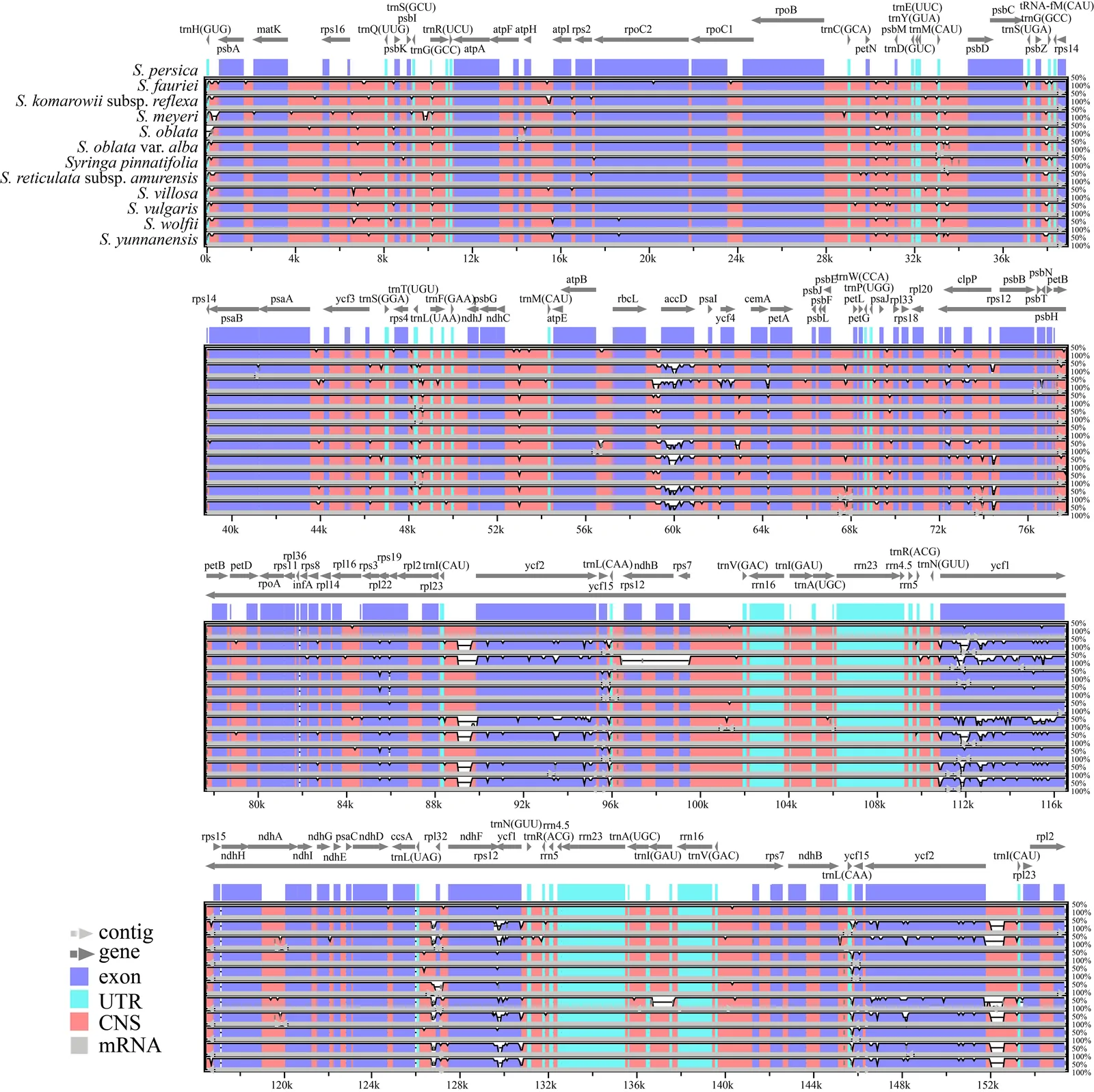
Visualization of the CPG comparison of 15 *Syringa* species using *S. pinnatifolia* as a reference. The horizontal axis represents the coordinates within the CPG, and the vertical axis indicates the percentage identity, ranging from 50% to 100%. The colors represent different regions: blue for exons, green for introns, and red for intergenic region.

**Figure 4 -:**
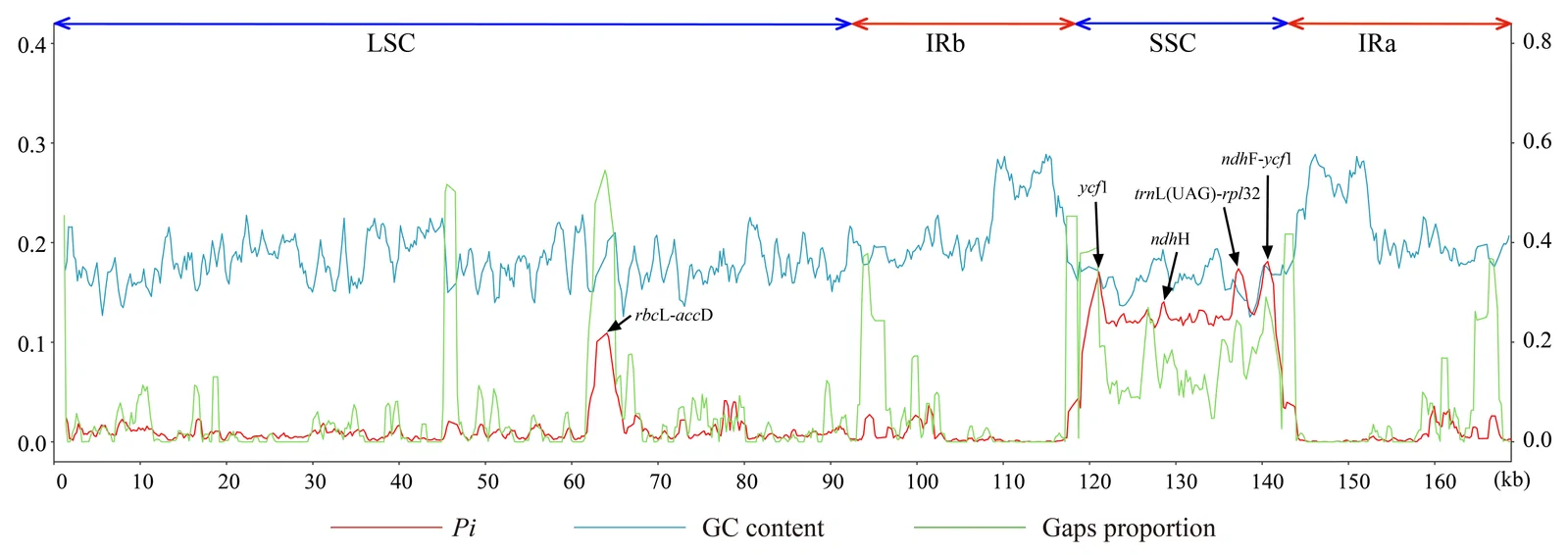
The nucleotide diversity (Pi), GC content, and gap proportion in the CPG sequences of 15 *Syringa* detected by sliding window analysis, using 600bp windows and a 200bp step size.

The CPGs of 15 *Syringa* species were visually compared with those of *S. pinnatifolia* as a reference using the mVISTA online database. The results indicated that the CPGs were generally conserved, although variations were present in both non-coding and coding regions (Figure 3). Regions exhibiting high sequence variation were mainly identified in the following intervals: *trn*H-*psb*A, *psb*I-*trn*G, *atp*H-*atp*I, *pet*N-*psb*M, *psb*C-*trn*S, *psb*Z-*trn*G, *rbc*L-*acc*D, *psb*E-*pet*L, *ycf*4-*cem*A, *trn*L-*rpl*32, *rpl*23-*ycf*2, and *ndh*F-*ycf*1 (Figure 3). Among the coding regions, *cem*A, *acc*D, *ndh*F, *rpl*22, *rps*3, *ycf*4, *clp*P, *ycf*1, and *ycf*2 exhibited the greatest variability. No major rearrangements or insertions were observed in any of the 15 CPGs (Figure 3). To further assess the DNA polymorphisms, mutation hotspot regions were screened using DnaSP (Figure 4). Pi values ranged from 0 to 0.07, reflecting a genome that was structurally conserved and compact, but contained highly variable regions. Subsequently, five mutation hotspots were identified as potential molecular markers. Among these, *ycf*1, *ndh*H, *trn*L-*rpl*32, and *ndh*F-*ycf*1 were located in the SSC region, and *rbc*L-*acc*D in the LSC region (Figure 4).

### Selection pressure analysis

**Figure 5 -:**
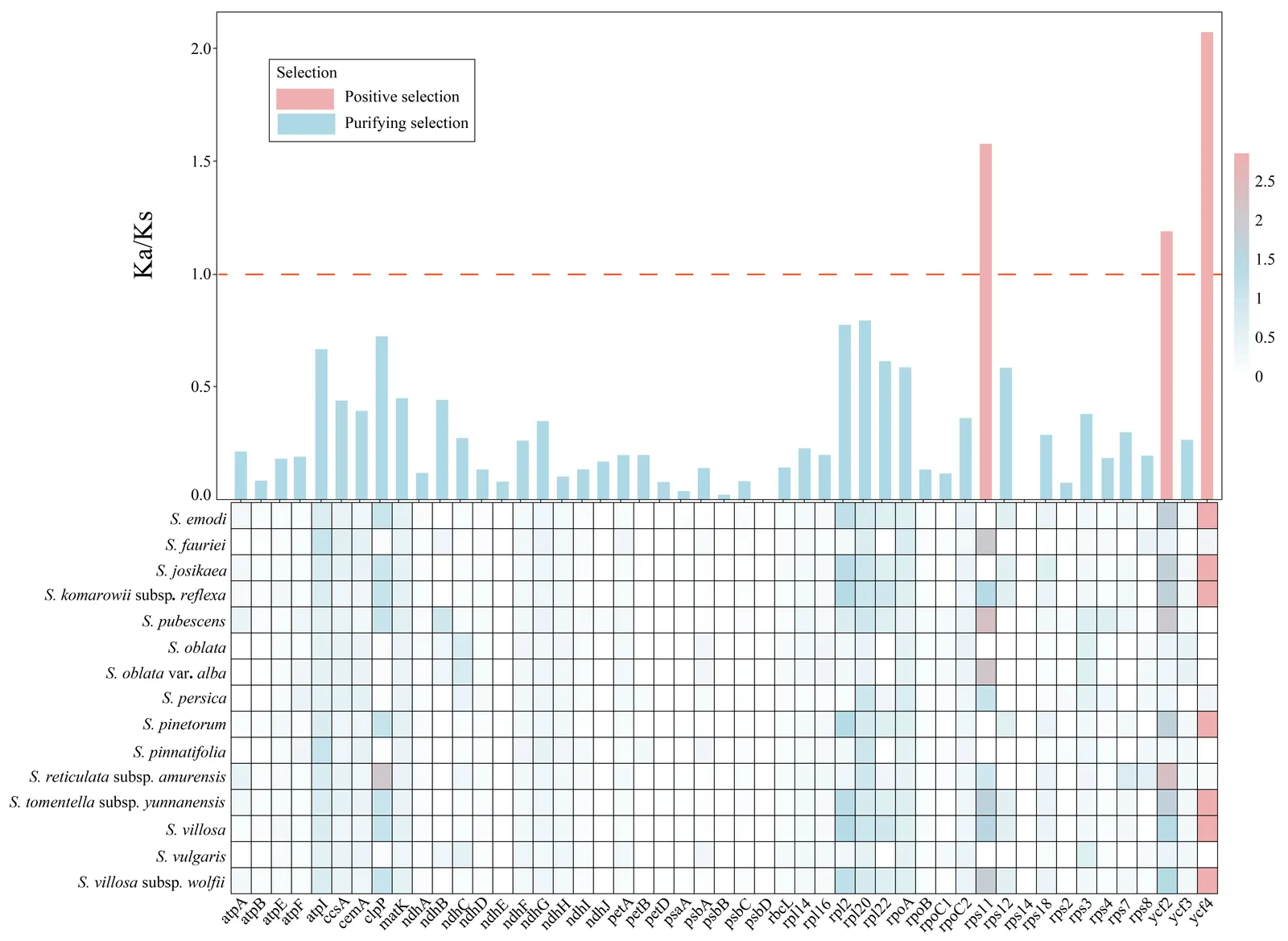
Results of the selective pressure analysis. (a) A bar chart showing the average Ka/Ks ratio for 49 CDS sequences across species, with two colors representing different types of selection: blue for purifying selection and orange for positive selection, with a baseline at Ka/Ks = 1. A heatmap of the 49 CDS sequences across 15 species, normalized with simple linear interpolation, where the color gradient from blue to white to orange corresponds to the magnitude of the Ka/Ks ratio.

To further investigate the selective pressures acting on PCGs, we performed Ka/Ks analysis using *S. pinnatifolia* as the reference species (Figure 5). After excluding the PCGs with a Ka/Ks ratio of NA, 49 genes were retained for further analysis. The results indicated that most genes of the 15 *Syringa* species had a Ka/Ks ratio < 1, suggesting that they had undergone purifying selection. However, three genes (*rps*11, *ycf*2, and *ycf*4) displayed Ka/Ks ratios significantly greater than one (1.5353, 1.1898, and 2.0750, respectively; Tables S3, S4), suggesting that they had experienced positive selection. Furthermore, these genes may have been critically involved in the adaptive evolution of *Syringa* species.

### Phylogenetic analysis and divergence time estimation

**Figure 6 -:**
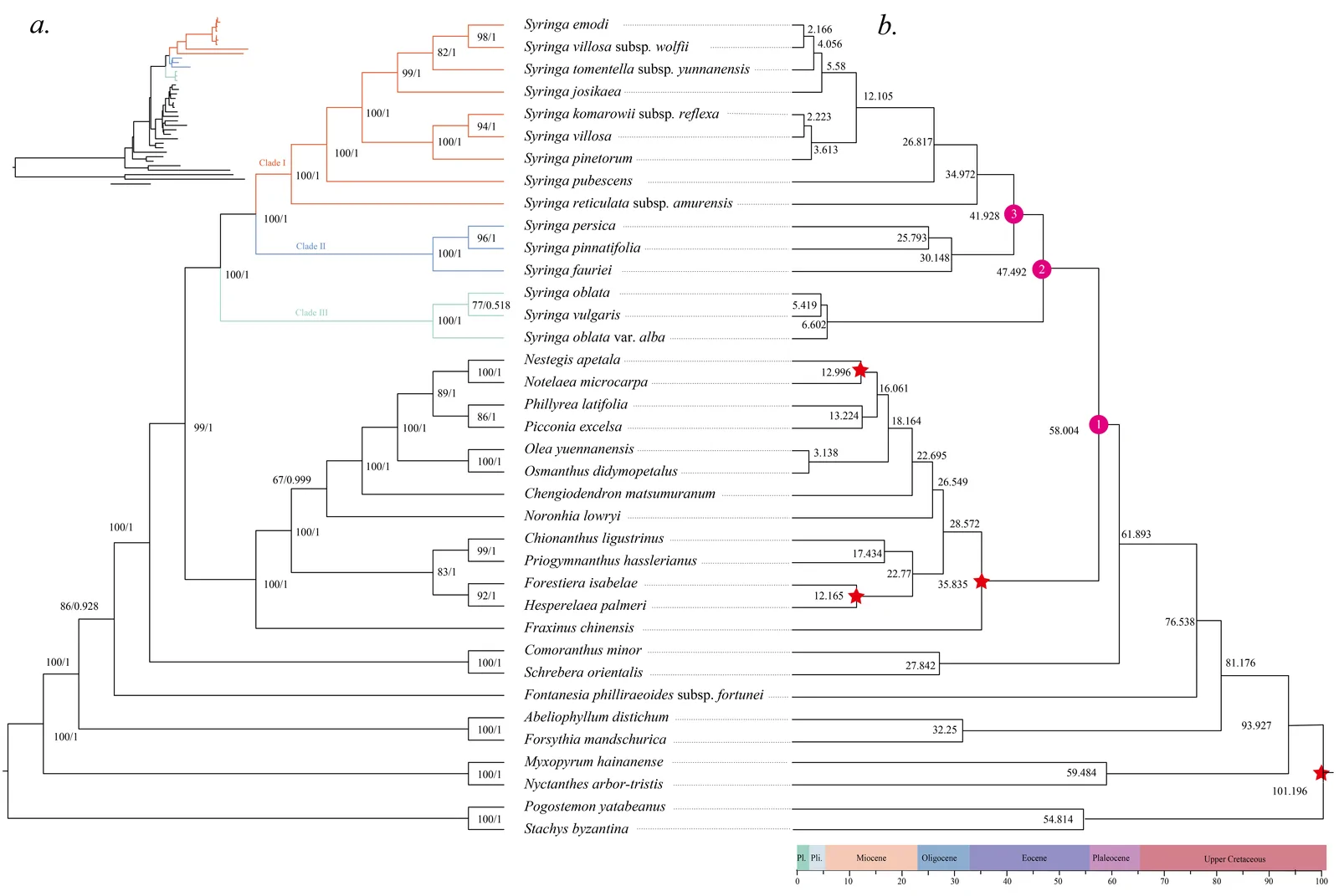
Phylogeny, divergence time estimate for *Syringa*. (a) Cladogram of the maximum likelihood (ML) phylogenetic tree based on 64 protein-coding genes (PCGs). All main clades in both trees (ML and BI) are identical. Node labels are marked with the bootstrap values and Bayesian posterior probabilities (bootstrap value/posterior probability). The upper left shows the ML tree with branch length. (b) Maximum clade-credibility tree (MCC tree) from BEAST. Node labels represent estimated divergence times in millions of years. Stars indicate time constraints in this analysis. Geological periods are marked with background colors. Mya: million years ago; Pli: Pliocene; Ple: Pleistocene.

In this study, we reconstructed the phylogenetic relationships of Oleaceae using ML and BI approaches based on PCGs and whole CPG sequences. The dataset included 32 Oleaceae accessions and two outgroup species (*Pogostemon yatabeanus* and *Stachys byzantina*). The resulting ML, BI, and BEAST MCC trees derived from PCGs showed highly consistent topologies (Figure 6). A final concatenated alignment of 64 PCGs comprising 54,948 sites was obtained after trimming poorly aligned regions and gaps containing missing genes (Table S5). Both ML and BI analyses yielded robust topologies, with most nodes showing high bootstrap support (BS > 90%) and posterior probability (PP = 1) across the datasets (Figure 6a ). The 15 *Syringa* species were resolved into three distinct clades: Clade I included *S. komarowii* subsp. *reflexa*, *S. villosa*, *S. villosa* subsp. *wolfii*, *S. tomentella* subsp. *yunnanensis*, *S. pubescens*, and *S. reticulata* subsp. *amurensis*; Clade II consisted of *S. fauriei*, *S. pinnatifolia*, and *S. persica* (BS = 100%; PP = 1); Clade III comprised *S. oblata* var. *alba*, *S. vulgaris*, and *S. oblata* (BS = 100%; PP = 1) (Figure 6 a , Figure S1a, and Figure S1b). The divergence times within *Syringa* were estimated using a PCG-based gene tree with time constraints. The divergence between *Syringa* and its sister group was dated back approximately 58 Mya. The crown age of all *Syringa* subclades was estimated to be approximately 47.49 Mya, indicating that the main diversification of the genus occurred during the Eocene (Figure 6b, Figure S2; Table 1).

**Table 1 -:** Estimated ages for *Syringa* subclades.

Subclade name	Mean age (Mya)	95% highest posterior density interval (HPD)
Subclade 1	58.004	42.79-73.85
Subclade 2	47.492	33.23-62.62
Subclade 3	41.928	27.69-55.27

## Discussion

The chloroplast is an essential organelle that plays crucial roles in the growth and development of plant cells (Zhang et al., 2024). In seed plants, the CPG generally ranges in size from 107 kb in Pinaceae to 170 kb in Geraniaceae, with the IR region typically spanning 20-30 kb (Zhang *et al*., 2012; Daniell et al., 2016). Most plant CPGs exhibit a conserved quadripartite structure, which consists of LSC and SSC regions separated by a pair of IRs (Shi et al., 2025; Wang et al., 2025). However, notable structural variations occur in certain lineages; for example, some species in the Fabaceae and Geraniaceae families have lost an entire IR region (Cui et al., 2024; Zhang *et al*., 2025). In the current study, comparative analysis revealed that the CPGs of the 15 *Syringa* species ranged from 154,019 (*S. pubescens*) to 158,020 bp (*S. komarowii* subsp. *reflexa*) (Figure 1; Table S2), placing them at the larger end of the size spectrum among the seed plant CPGs. All *Syringa* CPGs conformed to the typical quadripartite organization, comprising an LSC region (86,213-87,628 bp), an SSC region (17,239-21,180 bp), and two IR regions (24,468-25,897 bp). The GC content across the entire genome and within each structural region was highly conserved among *Syringa* species and consistent with the patterns observed in other vascular plants (Table S2).

The IR region is the most conserved structural component of the plant CPG (Yang et al., 2023), characterized by highly consistent length, organization, and boundary positions relative to SC regions. Expansions or contractions of the IR region are recognized as major contributors to overall genome size variation and represent the primary mechanism for the formation of pseudogenes (Yang *et al*., 2020a; Zhang et al., 2020 b ). In most plant species, such structural changes are reflected as minor positional shifts of the IR/SC boundaries near a limited set of conserved genes, which can occasionally lead to the pseudogenization of affected genes. The increased structural stability and higher GC content observed in IR regions have been attributed to mechanisms such as sequence conversion and enhanced DNA repair efficiency (Zhang *et al*., 2018; Yang *et al*., 2019; Xie et al., 2020). Previous studies have shown that chloroplast genes generally evolve slowly and maintain considerable sequence and structural conservation. IR expansions or contractions typically involve only minor changes, ranging from several to dozens of base pairs, and seldom alter the overall gene content (Kassem, 2025; Qin et al., 2025). Consistent with these observations, our analysis of 15 CPGs within the highly conserved genus *Syringa* revealed no evidence of large-scale IR expansion or contraction. This observation further supports the structural stability of this genomic region.

Previous phylogenetic studies of *Syringa* have primarily used nuclear ribosomal DNA Internal Transcribed Spacer (ITS) regions and Inter-Simple Sequence Repeats (ISSR) markers (Li et al., 2002; Yang et al., 2020 b ; Yao et al., 2021). However, very few phylogenetic studies have been based on CPG fragments. Highly variable regions in the CPG are particularly valuable for phylogenetics because they can be developed into molecular markers and aid in species delimitation (Yin et al., 2005; Xin et al., 2013; Yang *et al*., 2022; Ran et al., 2024). In the present study, we found that both the sequence and structure of the *Syringa* CPGs are highly conserved. mVISTA analysis revealed that most nucleotide variations occurred in non-coding regions, a pattern consistent with previous reports on angiosperms (Xing and Ree, 2017; Crepet et al., 2024; Cheng et al., 2025). Through Pi analysis, we identified five highly variable regions (Pi > 0.01), including two non-coding tRNA regions (*ycf*1 and *ndh*H) and three intergenic spacers (*trn*L-*rpl*32, *ndh*F-*ycf*1, and *rbc*L-*acc*D). These variable regions provide valuable genetic resources for future phylogenetic and phylogeographic studies on *Syringa*.

The evolutionary dynamics of substitution rates in cp genes have been increasingly clarified with the increasing availability of complete CPG sequences. Studies have generally indicated that non-synonymous substitution rates in photosynthesis-related genes differ significantly from those in housekeeping genes, reflecting stronger functional constraints on the photosynthetic machinery (Wu et al., 2017; Yan et al., 2019). Our findings are consistent with this pattern, as they show notably low Ka values for photosynthesis-related genes, further supporting the hypothesis that strong purifying selection acts on these genes (Figure 5). Although most plant cp genes evolve slowly under purifying selection, certain genes, particularly those involved in photosynthesis and metabolic pathways, show evidence of positive selection (Silva et al., 2023). In this study, we identified three genes, *rps*11, *ycf*2, and *ycf*4, with Ka/Ks ratios significantly greater than 1, suggesting that they have undergone positive selection. Among these, *rps*11 encodes a small subunit ribosomal protein involved in chloroplast translation, which is essential for the synthesis of photosynthetic components. *ycf*2, the largest chloroplast gene in angiosperms, encodes a motor protein that generates ATP for inner membrane translocation and is critical for cell survival (Kikuchi et al., 2018). *ycf*4 plays a key role in the assembly of photosystem I, and its dysfunction impairs photosynthetic electron transport. The widespread positive selection signature across diverse plant lineages (Chen et al., 2021; Yang et al., 2022) highlights its adaptive significance in various ecological contexts.

Plants CPGs are typically inherited maternally, resulting in a smaller effective population size than that of nuclear genomes. This characteristic leads to a shorter coalescence time of chloroplast haplotypes, making them particularly suitable for studying genetic variation and reconstructing the evolutionary history of chloroplasts (Guo et al., 2017; Zhang et al., 2024). Our study represents the first effort to establish a dated phylogeny for *Syringa* within Oleaceae using four fossil-based time constraints. We infer that the genus *Syringa* originated during the Oligocene and began to diversify during in the Eocene, followed by rapid radiation throughout the Pliocene and Pleistocene (Figure 6 b ). The Pliocene-Pleistocene period appears to play a crucial role in the speciation of *Syringa*, a pattern of rapid diversification also observed across numerous other East Asian plant groups (Xing and Ree, 2017; Zhao et al., 2022). This burst of speciation was likely driven by Pleistocene glacial-interglacial cycles and the associated climatic fluctuations. East Asia, which has been less severely affected by Quaternary glaciations, may have provided refugia to various plant groups that reduced extinction rates and promoted speciation (Axelrod et al., 1998; Yan et al., 2018; Qian and Ricklefs, 2000). The divergence timescale proposed in this study provides a foundational temporal framework for future ecological, evolutionary, and biogeographic studies of *Syringa,* an economically and ecologically significant genus. Although this study represents the first attempt to explore the phylogeny and divergence times of the genus *Syringa* based on 15 species, the limited sample size imposes certain constraints on the findings. Moreover, the selected species may not fully represent the diversity within the genus, which could affect the generalizability and accuracy of the inferred phylogenetic relationships and divergence time estimates. Future studies should incorporate a broader range of *Syringa* species to enable more comprehensive and systematic investigations.

## Conclusions

In the present study, we analyzed the complete CPGs of 15 *Syringa* species. All examined genomes exhibited a typical angiosperm quadripartite structure and contained 130-132 genes, including 85-88 PCGs, 35-37 tRNAs, and eight rRNAs. Genome organization was highly conserved, with the highest sequence variation observed in the SSC region compared with the IR regions. No major IR expansions, contractions, genomic rearrangements, or insertions were detected in these 15 CPGs. We identified five highly variable regions suitable for species delimitation within this genus. In addition, three genes (*rps*11, *ycf*2, and *ycf*4) showed signatures of positive selection, suggesting their critical roles in the adaptive evolution of *Syringa*. Phylogenetic analysis based on complete CPGs confirmed the monophyly of the 15 *Syringa* species, which were resolved into three distinct clades. Molecular dating placed the divergence of *Syringa* from its sister genus at approximately 58 Mya during the Eocene, with initial diversification beginning around 47.49 Mya during the Oligocene. Overall, our findings provide a robust genomic framework and reliable divergence timescale for future research on *Syringa*. Our study also supports *Syringa* species identification and will facilitate further ecological, evolutionary, and applied studies of this economically and ecologically important genus.

## Supplementary material

The following online material is available for this article:

Figure S1 - Phylogenetic tree based on 64 protein-coding genes (PCGs).

Figure S2 - Maximum clade-credibility tree (MCC tree) from BEAST.

Table S1 - Species and data description of CPGs used in this study.

Table S2 - General information and comparison of chlorplast genomes of 15 *Syringa* species.

Table S3 - The average Ka/Ks ratio for 49 gene sequences across 15 *Syringa* species in this study.

Table S4 - Results of the selective pressure analysis.

Table S5 - Comparison of protein-coding genes.

## Data Availability

The three newly assembled and annotated CPGs have been submitted to the Genome Sequence Archive in National Genomics Data Center, China National Center for Bioinformation (https://ngdc.cncb.ac.cn/genbase), with accession numbers listed in Table S1. Other data generated or analyzed in our study are included in the supplementary files.
