# Supplementary material for: Chloroplast genome comparative analysis and phylogenetic relationships of 15 Syringa species (Oleaceae)
Source: Genet Mol Biol. 2026 Sep 7;49(3):e20260016. doi: 10.1590/1678-4685-GMB-2026-0016 (PMC13555786; doi:10.1590/1678-4685-GMB-2026-0016)
Supplement: Supplementary file 1 [file 1415-4757-GMB-49-3-e20260016-s1.pdf]

Genetics and Molecular Biology
https://doi.org/10.1590/1678-4685-GMB-2026-0016

Supplementary Material to "Chloroplast genome comparative analysis and phylogenetic relationships of 15 *Syringa* species (Oleaceae)"

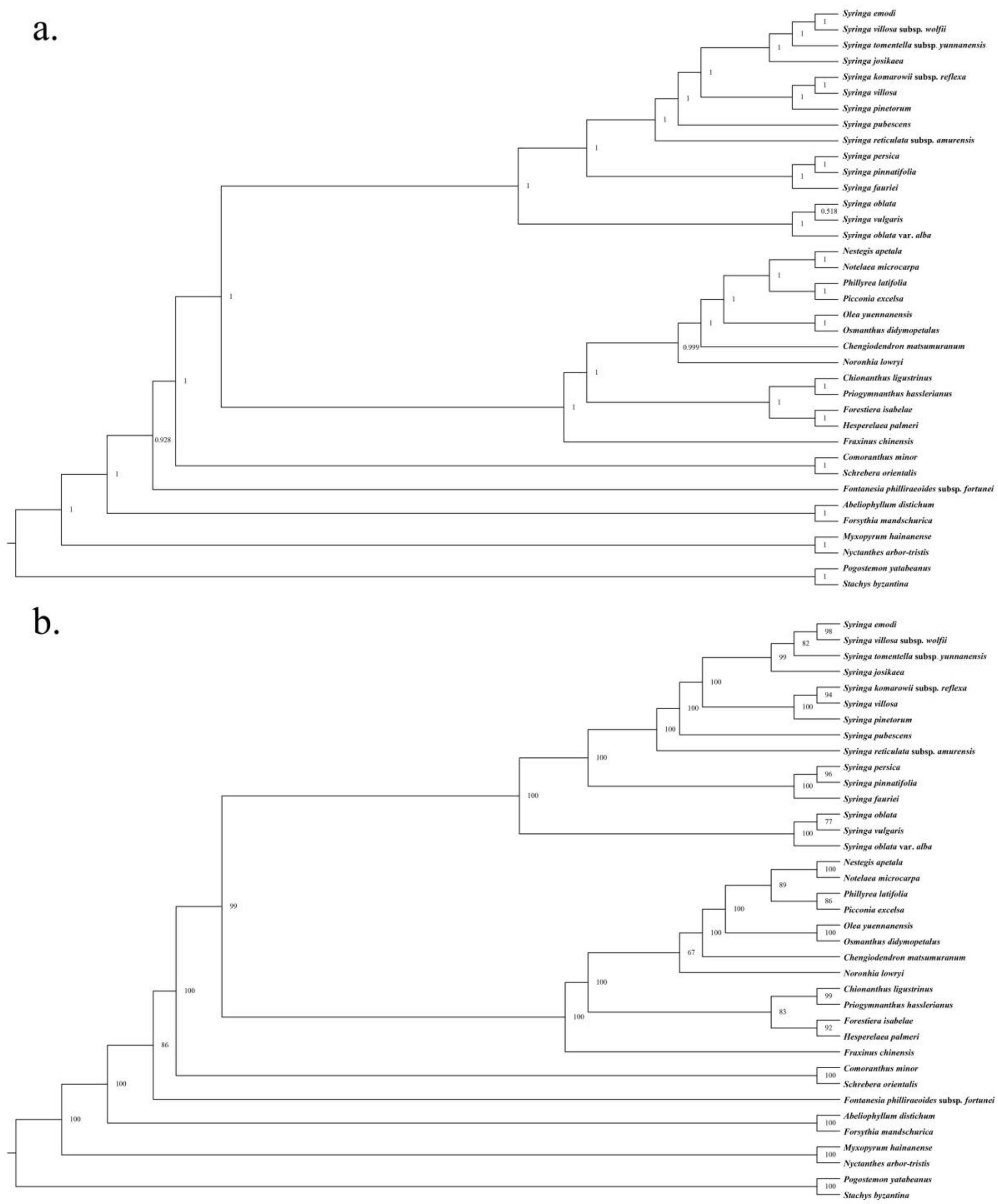

Figure S1 - Phylogenetic tree based on 64 protein-coding genes (PCGs). All main clades in both trees (ML and BI) are identical. Node labels are marked with the bootstrap values and Bayesian posterior probabilities (bootstrap value/posterior probability).
