# Supplementary material for: Chloroplast genome comparative analysis and phylogenetic relationships of 15 Syringa species (Oleaceae)
Source: Genet Mol Biol. 2026 Sep 7;49(3):e20260016. doi: 10.1590/1678-4685-GMB-2026-0016 (PMC13555786; doi:10.1590/1678-4685-GMB-2026-0016)
Supplement: Supplementary file 3 [file 1415-4757-GMB-49-3-e20260016-s3.pdf]

Genetics and Molecular Biology
https://doi.org/10.1590/1678-4685-GMB-2026-0016

Supplementary Material to "Chloroplast genome comparative analysis and phylogenetic relationships of 15 *Syringa* species (Oleaceae)"

Table S1 - Species and data description of CPGs used in this study.

Source	Taxon	Longitude	Latitude	Altitude (m)	Collection site	Collection number	Herbarium deposit*	Genbank accession
Assembled in our lab	*Syringa pinnatifolia*	105.8892	38.5093	1799	Yinchuan, Ningxia	zlnmu2024085	NMU	C_AA122236
	Syringa vulgaris	106.0986	38.4983	1084	Yinchuan, Ningxia	zlnmu2021150	NMU	C_AA122238
	Syringa pubescens	40.0023	116.208	78	Beijing,Hai dianqu	zlnum2025100	NMU	C_AA122237
Downloaded from Genbank	*Syringa × persica*					-	-	NC_042280
	Syringa reticulata subsp. *amurensis*					-	-	MW525283
	Syringa fauriei					-	-	OR209257
	Syringa oblata					-	-	MW464119
	Syringa oblata var. *alba*					-	-	ON920512

Source	Taxon	Longitude	Latitude	Altitude (m)	Collection site	Collection number	Herbarium deposit*	Genbank accession
	Syringa villosa subsp. *wolfii*					-	-	MN901631
	Syringa villosa					-	-	NC_061378
	Syringa tomentella subsp. *yunnanensis*					-	-	NC_042468
	Syringa komarowii subsp. *reflexa*					-	-	MT648823
	Syringa pinetorum					-	-	OP531898
	Syringa emodi					-	-	PZ239408
	Syringa josikaea					-	-	OP531896
	Picconia excelsa					-	-	NC_042466
	Phillyrea latifolia					-	-	NC_042465
	Notelaea microcarpa					-	-	NC_042459
	Nestegis apetala					-	-	NC_036983
	Osmanthus didymopetalus					-	-	NC_057502
	Chengiodendron matsumuranum					-	-	NC_072069

Source	Taxon	Longitude	Latitude	Altitude (m)	Collection site	Collection number	Herbarium deposit*	Genbank accession
	Chionanthus ligustrinus					-	-	NC_042284
	Olea yuennanensis					-	-	NC_086553
	Noronhia lowryi					-	-	NC_036984
	Forestiera isabelae					-	-	NC_036981
	Hesperelaea palmeri					-	-	LN515489
	Priogymnanthus hasslerianus					-	-	NC_042279
	Schrebera orientalis					-	-	NC_042266
	Comoranthus minor					-	-	MH817901
	Fraxinus chinensis					-	-	PQ456186
	Abeliophyllum distichum					-	-	NC_031445
	Forsythia mandschurica					-	-	NC_048504
	Myxopyrum hainanense					-	-	MN908148
	Fontanesia philliraeoides subsp. *fortunei*					-	-	MG255754

Abbreviations: NMU, Herbarium of North MinZu University.
