# Supplementary material for: Chloroplast genome comparative analysis and phylogenetic relationships of 15 Syringa species (Oleaceae)
Source: Genet Mol Biol. 2026 Sep 7;49(3):e20260016. doi: 10.1590/1678-4685-GMB-2026-0016 (PMC13555786; doi:10.1590/1678-4685-GMB-2026-0016)
Supplement: Supplementary file 4 [file 1415-4757-GMB-49-3-e20260016-s4.pdf]

Genetics and Molecular Biology
https://doi.org/10.1590/1678-4685-GMB-2026-0016

Supplementary Material to "Chloroplast genome comparative analysis and phylogenetic relationships of 15 *Syringa* species (Oleaceae)"

Table S2 - General information and comparison of chlorplast genomes of 15 *Syringa* species.

species	total (bp)	Total GC%	LSC (bp)	LSC GC%	SSC (bp)	SSC GC%	IR (bp)	IR GC%	CDS	rRNA	tRNA	total genes
Syringa pinnatifolia	155366	0.379	86248	0.359	17772	0.321	25673	0.432	88	8	35	131
Syringa × persica	155414	0.38	86237	0.36	17761	0.322	25708	0.432	88	8	35	131
Syringa reticulata subsp. *amurensis*	156141	0.38	87108	0.361	17239	0.329	25897	0.429	87	8	37	132
Syringa fauriei	155421	0.379	86278	0.359	17743	0.322	25700	0.432	85	8	37	130
Syringa oblata	155668	0.379	86276	0.359	17926	0.321	25733	0.432	88	8	36	132
Syringa vulgaris	156600	0.379	86642	0.361	19118	0.325	25420	0.434	88	8	35	131
Syringa oblata var. *alba*	155648	0.379	86247	0.359	17937	0.321	25732	0.432	88	8	36	132
Syringa villosa subsp. *wolfii*	156517	0.38	86684	0.311	19109	0.326	25362	0.431	87	8	36	131

species	total (bp)	Total GC%	LSC (bp)	LSC GC%	SSC (bp)	SSC GC%	IR (bp)	IR GC%	CDS	rRNA	tRNA	total genes
Syringa villosa	156736	0.38	86912	0.361	19094	0.327	25365	0.431	87	8	36	131
Syringa tomentella subsp. *yunnanensis*	156822	0.38	86651	0.361	19047	0.327	25562	0.43	88	8	35	131
Syringa komarowii subsp. *reflexa*	158020	0.379	87628	0.362	19040	0.326	25676	0.429	88	8	36	132
Syringa pubescens	154019	0.38	86213	0.361	17714	0.325	25046	0.434	88	8	35	131
Syringa pinetorum	157131	0.379	86872	0.361	19245	0.326	25507	0.43	85	8	37	130
Syringa josikaea	156851	0.379	86641	0.361	19072	0.326	25569	0.429	85	8	37	130
Syringa emodi	156687	0.38	86716	0.361	19109	0.325	25431	0.431	85	8	37	130
