# Supplementary material for: Chloroplast genome comparative analysis and phylogenetic relationships of 15 Syringa species (Oleaceae)
Source: Genet Mol Biol. 2026 Sep 7;49(3):e20260016. doi: 10.1590/1678-4685-GMB-2026-0016 (PMC13555786; doi:10.1590/1678-4685-GMB-2026-0016)
Supplement: Supplementary file 5 [file 1415-4757-GMB-49-3-e20260016-s5.pdf]

Genetics and Molecular Biology
https://doi.org/10.1590/1678-4685-GMB-2026-0016

Supplementary Material to "Chloroplast genome comparative analysis and phylogenetic relationships of 15 *Syringa* species (Oleaceae)"

Table S3 - The average Ka/Ks ratio for 49 gene sequences across 15 *Syringa* species in this study.

gene	*Syringa emodi*	*S. fauriei*	*S. josikaea*	*S. komarowii subsp. reflexa*	*S. pubescens*	*S. oblata*	*S. oblata var. alba*	*S. persica*	*S. pinetorum*	*S. pinnatifolia*	*S. reticulata subsp. amurensis*	*S. tomentella subsp. yunnanensis*	*S. villosa*	*S. vulgaris*	*S. villosa subsp. wolfii*
*atp*A	0.261	0.047	0.261	0.196	0.436	0	0	0.052	0.135	0.052	0.460	0.261	0.135	0	0.261
*atp*B	0.116	0.078	0.116	0.107	0.089	0.032	0.064	0.025	0.132	0.035	0.097	0.116	0.107	0.032	0.116
*atp*E	0.170	0.128	0.170	0.256	0.128	0.170	0.170	0.170	0.256	0.170	0.197	0.170	0.256	0.170	0.170
*atp*F	0.142	0.142	0.142	0.142	0.340	0.142	0.286	0.416	0.094	0.286	0.134	0.142	0.142	0.142	0.142
*atp*I	0.681	1.018	0.681	0.681	0.509	0.509	0.509	0.505	0.681	1.018	0.703	0.681	0.681	0.509	0.681
*ccs*A	0.455	0.590	0.486	0.358	0.430	0.467	0.467	0.520	0.333	0.316	0.462	0.486	0.358	0.421	0.455
*cem*A	0.357	0.541	0.404	0.304	0.251	0.428	0.428	0.541	0.357	0.476	0.314	0.404	0.357	0.393	0.357
*clp*P	1.052	0.095	0.973	1.058	1.043	0.097	0.097	0.072	1.058	0.058	2.078	1.014	1.058	0.097	1.014
*mat*K	0.501	0.429	0.501	0.497	0.545	0.377	0.377	0.417	0.497	0.425	0.424	0.405	0.497	0.352	0.501
*ndh*A	0.100	0.118	0.100	0.125	0.100	0.161	0.100	0.125	0.149	0.124	0.146	0.100	0.125	0.099	0.100
*ndh*B	0	0.327	0	0	0.904	0.327	0.327	0	0	0	0	0	0	0.326	0
*ndh*C	0.134	0.081	0.134	0.134	0.169	0.755	0.770	0.384	0.134	0.124	0.341	0.134	0.134	0.507	0.134
*ndh*D	0.110	0.111	0.136	0.127	0.158	0.139	0.155	0.176	0.140	0.110	0.162	0.120	0.112	0.111	0.110
*ndh*E	0	0	0	0	0	0	0	0	0.077	0	0	0	0	0	0
*ndh*F	0.223	0.242	0.223	0.246	0.276	0.290	0.266	0.285	0.267	0.300	0.278	0.209	0.246	0.322	0.223

gene	*Syringa emodi*	*S. fauriei*	*S. josikaea*	*S. komarowii subsp. reflexa*	*S. pubescens*	*S. oblata*	*S. oblata var. alba*	*S. persica*	*S. pinetorum*	*S. pinnatifolia*	*S. reticulata subsp. amurensis*	*S. tomentella subsp. yunnanensis*	*S. villosa*	*S. vulgaris*	*S. villosa subsp. wolfii*
*ndh*G	0.369	0.397	0.322	0.322	0.397	0.274	0.346	0.346	0.322	0.474	0.346	0.322	0.322	0.273	0.369
*ndh*H	0.223	0.242	0.223	0.246	0.276	0.290	0.266	0.285	0.267	0.300	0.278	0.209	0.246	0.322	0.223
*ndh*I	0.100	0.143	0.100	0.081	0.190	0.143	0.143	0.143	0.085	0.220	0.193	0.100	0.100	0.143	0.100
*ndh*J	0	0	0	0	0.166	0	0	0	0	0	0	0	0	0	0
*pet*A	0.161	0.307	0.161	0.161	0.238	0.211	0.241	0.206	0.161	0.162	0.203	0.161	0.161	0.241	0.161
*pet*B	0	0	0	0	0	0	0	0.159	0	0.232	0	0	0	0	0
*pet*D	0.077	0	0.077	0	0	0	0	0	0	0	0	0.077	0	0	0.077
*psa*A	0.058	0.038	0.058	0.036	0.015	0.022	0.021	0.024	0.036	0.021	0.038	0.058	0.036	0.022	0.058
*psb*A	0.073	0.156	0.073	0.073	0.034	0.294	0.294	0.146	0.073	0.294	0.058	0.073	0.073	0.294	0.073
*psb*B	0.022	0.016	0.021	0.021	0	0	0	0	0.021	0	0.021	0.022	0.021	0	0.022
*psb*C	0.095	0.071	0.084	0.079	0.095	0.049	0.049	0.064	0.079	0.094	0.126	0.095	0.079	0.049	0.095
*psb*D	0	0	0	0	0	0	0	0	0	0	0	0	0	0	0
*rbc*L	0.124	0.095	0.186	0.124	0.121	0.188	0.182	0.089	0.124	0.133	0.151	0.124	0.124	0.227	0.124
*rpl*14	0.206	0.196	0.206	0.206	0.358	0.275	0.275	0.131	0.206	0.178	0.281	0.206	0.206	0.275	0.206
*rpl*16	0.177	0.141	0.177	0.177	0.172	0.309	0.309	0.105	0.241	0.178	0.243	0.177	0.177	0.219	0.177
*rpl*20	1.217	0.223	1.306	1.391	0.676	0.252	0.252	0.126	1.391	0.250	0.371	1.306	1.391	0.252	1.217
*rpl*22	0.805	0.683	0.961	0.960	0.917	0.412	0.412	0.982	0.805	0.981	0.917	0.805	0.960	0.497	0.805
*rpl*2	0.613	0	0.613	0.921	0.614	0	0	0.306	0.613	0	0.306	0.613	0.921	0	0.613
*rpo*A	0.628	0.732	0.637	0.628	0.415	0.514	0.514	0.714	0.628	0.520	0.466	0.637	0.628	0.514	0.628
*rpo*B	0.133	0.097	0.138	0.142	0.164	0.140	0.119	0.099	0.142	0.130	0.139	0.133	0.148	0.113	0.133
*rpo*C1	0.063	0.135	0.063	0.081	0.193	0.170	0.189	0.109	0.081	0.101	0.109	0.063	0.081	0.214	0.063
*rpo*C2	0.384	0.285	0.384	0.352	0.391	0.416	0.381	0.357	0.343	0.289	0.350	0.371	0.352	0.388	0.384
*rps*11	0	1.873	0	1.246	2.229	0	1.781	0.900	0	0	1.358	1.317	0	0	1.577
*rps*12	0.585	0	0.585	0.585	0	0	0	0	0.585	0	0	0.585	0.585	0	0.585

gene	*Syringa emodi*	*S. fauriei*	*S. josikaea*	*S. komarowii subsp. reflexa*	*S. pubescens*	*S. oblata*	*S. oblata var. alba*	*S. persica*	*S. pinetorum*	*S. pinnatifolia*	*S. reticulata subsp. amurensis*	*S. tomentella subsp. yunnanensis*	*S. villosa*	*S. vulgaris*	*S. villosa subsp. wolfii*
*rps*14	0	0	0	0	0	0	0	0	0	0	0	0	0	0	0
*rps*18	0.394	0.056	0.602	0.394	0.263	0.129	0.129	0.088	0.394	0.129	0.391	0.394	0.394	0.129	0.394
*rps*2	0.050	0.063	0.050	0.050	0.162	0.043	0.043	0.174	0.050	0.144	0.075	0.050	0.050	0.043	0.050
*rps*3	0.261	0.188	0.261	0.261	0.645	0.618	0.618	0.512	0.261	0.344	0.304	0.261	0.261	0.618	0.261
*rps*4	0.139	0.184	0.139	0.139	0.644	0.126	0.126	0.320	0.110	0.138	0.136	0.139	0.139	0.126	0.139
*rps*7	0.237	0	0.237	0.237	0.352	0	0	0	0.237	0	0.669	0.237	0.237	0	0.237
*rps*8	0.142	0.415	0.071	0.057	0.091	0.195	0.282	0.281	0.057	0.281	0.570	0.071	0.057	0.195	0.142
*ycf*2	1.730	0.403	1.744	1.712	1.974	0.389	0.351	0.353	1.712	0.269	2.281	1.730	1.356	0.395	1.448
*ycf*3	0.241	0.081	0.241	0.241	0.241	0.482	0.482	0	0.241	0.241	0.241	0.241	0.241	0.241	0.241
*ycf*4	2.856	0.305	2.856	2.856	0	0	0	0.304	2.856	0	0.148	2.856	2.856	0	2.856
