# Supplementary material for: Chloroplast genome comparative analysis and phylogenetic relationships of 15 Syringa species (Oleaceae)
Source: Genet Mol Biol. 2026 Sep 7;49(3):e20260016. doi: 10.1590/1678-4685-GMB-2026-0016 (PMC13555786; doi:10.1590/1678-4685-GMB-2026-0016)
Supplement: Supplementary file 6 [file 1415-4757-GMB-49-3-e20260016-s6.pdf]

Genetics and Molecular Biology
https://doi.org/10.1590/1678-4685-GMB-2026-0016

Supplementary Material to "Chloroplast genome comparative analysis and phylogenetic relationships of 15 *Syringa* species (Oleaceae)"

Table S4 - Results of the selective pressure analysis.

Type	gene	Ka	Ks	Ka/Ks	Length
ATP synthase	*atp*A	0.0034	0.0152	0.2130	1521
ATP synthase	*atp*B	0.0024	0.0293	0.0841	1494
ATP synthase	*atp*E	0.0072	0.0397	0.1835	399
ATP synthase	*atp*F	0.0035	0.0195	0.1892	552
ATP synthase	*atp*I	0.0064	0.0100	0.6694	741
Cytochrome b/f complex	*pet*A	0.0045	0.0233	0.1961	960
Cytochrome b/f complex	*pet*B	0.0062	0.0327	0.1955	645
Cytochrome b/f complex	*pet*D	0.0028	0.0356	0.0772	480
NADPH dehydrogenase	*ndh*A	0.0054	0.0464	0.1183	1083
NADPH dehydrogenase	*ndh*B	0.0013	0.0028	0.4425	1530
NADPH dehydrogenase	*ndh*C	0.0078	0.0398	0.2710	360
NADPH dehydrogenase	*ndh*D	0.0083	0.0638	0.1318	1493
NADPH dehydrogenase	*ndh*E	0.0041	0.0531	0.0774	303
NADPH dehydrogenase	*ndh*F	0.0259	0.1038	0.2598	2208
NADPH dehydrogenase	*ndh*G	0.0143	0.0417	0.3466	528
NADPH dehydrogenase	*ndh*H	0.0048	0.0491	0.1001	1179
NADPH dehydrogenase	*ndh*I	0.0073	0.0588	0.1321	509
NADPH dehydrogenase	*ndh*J	0.0053	0.0319	0.1663	474
Other genes	*ccs*A	0.0138	0.0320	0.4401	969
Other genes	*cem*A	0.0153	0.0401	0.3942	687
Other genes	*clp*P	0.0579	0.0599	0.7241	588
Other genes	*mat*K	0.0160	0.0359	0.4498	1518
Other genes	*ycf*2	0.0152	0.0111	1.1897	5895
Other genes	*ycf*3	0.0028	0.0117	0.2638	504
Other genes	*ycf*4	0.0164	0.0109	2.0749	552
Photosystem	*psa*A	0.0011	0.0295	0.0359	2250
Photosystem	*psb*A	0.0012	0.0139	0.1386	1059
Photosystem	*psb*B	0.0009	0.0417	0.0206	1524
Photosystem	*psb*C	0.0018	0.0219	0.0803	1419
Photosystem	*psb*D	0	0.0260	0	1059
Ribosomal proteins	*rbc*L	0.0026	0.0188	0.1410	1431
Ribosomal proteins	*rpl*14	0.0103	0.0466	0.2274	366
Ribosomal proteins	*rpl*16	0.0099	0.0524	0.1985	405
Ribosomal proteins	*rpl*2	0.0032	0.0052	0.6131	822

Type	gene	Ka	Ks	Ka/Ks	Length
Ribosomal proteins	*rpl*20	0.0209	0.0292	0.7746	384
Ribosomal proteins	*rpl*22	0.0226	0.0323	0.7936	470
Ribosomal proteins	*rps*11	0.0161	0.0102	1.5353	414
Ribosomal proteins	*rps*12	0.0070	0.0120	0.5853	369
Ribosomal proteins	*rps*14	0	0.0147	0	300
Ribosomal proteins	*rps*18	0.0096	0.0369	0.2856	303
Ribosomal proteins	*rps*2	0.0040	0.0587	0.0730	708
Ribosomal proteins	*rps*3	0.0131	0.0380	0.3782	654
Ribosomal proteins	*rps*4	0.0052	0.0305	0.1829	603
Ribosomal proteins	*rps*7	0.0038	0.0126	0.2975	465
Ribosomal proteins	*rps*8	0.0049	0.0356	0.1937	402
RNA polymerase	*rpo*A	0.0208	0.0361	0.5868	1010
RNA polymerase	*rpo*B	0.0057	0.0431	0.1315	3210
RNA polymerase	*rpo*C1	0.0031	0.0298	0.1142	2061
RNA polymerase	*rpo*C2	0.0114	0.0317	0.3619	4175
