# Supplementary material for: Chloroplast genome comparative analysis and phylogenetic relationships of 15 Syringa species (Oleaceae)
Source: Genet Mol Biol. 2026 Sep 7;49(3):e20260016. doi: 10.1590/1678-4685-GMB-2026-0016 (PMC13555786; doi:10.1590/1678-4685-GMB-2026-0016)
Supplement: Supplementary file 7 [file 1415-4757-GMB-49-3-e20260016-s7.pdf]

Genetics and Molecular Biology
https://doi.org/10.1590/1678-4685-GMB-2026-0016

Supplementary Material to "Chloroplast genome comparative analysis and phylogenetic relationships of 15 *Syringa* species (Oleaceae)"

Table S5 - Comparison of protein-coding genes.

gene	Total site num	Pi site num	Percent of Pi site (%)
*atp*A	1521	160	10.519
*atp*E	399	49	12.281
*atp*F	573	89	15.532
*atp*H	243	14	5.761
*atp*I	741	56	7.557
*clp*P	588	184	31.293
*mat*K	1590	523	32.893
*ndh*A	1095	160	14.612
*ndh*B	1560	56	3.59
*ndh*C	360	40	11.111
*ndh*D	1527	292	19.122
*ndh*E	303	37	12.211
*ndh*F	2592	922	35.571
*ndh*G	528	69	13.068
*ndh*H	1182	162	13.706
*ndh*I	552	122	22.101
*ndh*J	492	62	12.602
*pet*A	966	106	10.973
*pet*B	657	71	10.807
*pet*D	480	39	8.125
*pet*G	111	6	5.405
*pet*L	93	10	10.753
*pet*N	87	6	6.897
*psa*A	2250	151	6.711
*psa*C	243	24	9.877
*psb*A	1059	76	7.177
*psb*B	1524	148	9.711
*psb*C	1419	99	6.977
*psb*D	1059	65	6.138
*psb*E	249	16	6.426
*psb*F	117	6	5.128
*psb*H	243	49	20.165
*psb*I	168	67	39.881
*psb*J	120	7	5.833

**gene**	**Total site num**	**Pi site num**	**Percent of Pi site (%)**
*psb*K	201	32	15.92
*psb*L	114	4	3.509
*psb*M	150	55	36.667
*psb*N	129	6	4.651
*psb*T	114	22	19.298
*rbc*L	1479	208	14.064
*rpl*14	366	38	10.383
*rpl*16	408	72	17.647
*rpl*2	945	553	58.519
*rpl*20	387	72	18.605
*rpl*23	315	55	17.46
*rpl*32	180	60	33.333
*rpl*33	198	31	15.657
*rpl*36	111	11	9.91
*rpo*B	3213	344	10.707
*rpo*C1	2088	289	13.841
*rpo*C2	4245	920	21.673
*rps*11	414	55	13.285
*rps*14	300	28	9.333
*rps*15	270	51	18.889
*rps*16	273	53	19.414
*rps*18	378	205	54.233
*rps*2	840	229	27.262
*rps*3	672	128	19.048
*rps*4	603	62	10.282
*rps*7	465	14	3.011
*rps*8	411	65	15.815
*ycf*2	7296	3213	44.038
*ycf*3	504	26	5.159
*ycf*4	588	157	26.701
